# The Impact of Additive and Subtractive Manufacturing on the Adhesion and Durability of Titanium–Zirconia Restorative Materials

**DOI:** 10.3390/jfb16070257

**Published:** 2025-07-11

**Authors:** Omar Alageel, Najm Alfrisany, Abdullah Alshamrani, Omar Alsadon

**Affiliations:** Dental Health Department, College of Applied Medical Sciences, King Saud University, P.O. Box 10219, Riyadh 11433, Saudi Arabiaabalshamrani@ksu.edu.sa (A.A.); oalsadon@ksu.edu.sa (O.A.)

**Keywords:** titanium, zirconia, CAD/CAM, 3D printing, shear bond strength, digital dentistry

## Abstract

This study aimed to investigate the bonding strength and durability of titanium alloys bonded to zirconia-based materials produced using subtractive and additive digital methods. Two titanium alloy groups (N = 20) and two zirconia ceramic groups (N = 60) were fabricated using CAD/CAM milling from prefabricated discs (Ti-ML and Zr-ML), and 3D printing via SLM (Ti-3D) and DLP/LCM systems (Zr-3D). The specimens were bonded with dental cement to form four test groups: Zr-ML/Ti-ML, Zr-ML/Ti-3D, Zr-3D/Ti-ML, and Zr-3D/Ti-3D. Half of the specimens in each group underwent thermocycling to assess the effect of aging on bond strength. The density, microhardness, and surface morphology were evaluated, along with the shear bond strength and failure modes of the resin composites. Statistical differences were analyzed using one-way ANOVA and Tukey’s HSD test across all groups. The 3D-printed specimens of both materials exhibited higher microhardness and lower surface roughness than the milled specimens. The shear bond strength (SBS) was the highest in the Ti-ML/Zr-ML combination group before and after thermocycling, which had more cohesive failures, whereas the lowest bond strength was observed in the Ti-3D/Zr-ML group. The adhesion between titanium and zirconia-based materials was the strongest when both were fabricated using subtractive methods, followed by additive and mixed-method combinations.

## 1. Introduction

Titanium alloys are essential materials in dentistry, used for manufacturing various dental prostheses and restorations [1,2,3,4]. Their popularity stems from their excellent mechanical strength, superior corrosion resistance, remarkable biocompatibility with human tissue, and ability to osseointegrate [1,2,5]. In prosthodontics, titanium alloys are utilized in dental implants, including the root and abutment of the implant-supported crowns or bridge, and in frameworks for removable partial dentures and implant-supported dentures [2,5,6]. Zirconia, or zirconium dioxide (ZrO_2_), is a highly durable and biocompatible ceramic widely used in dentistry for fixed restorations such as crowns, bridges, implant abutments, inlays, and onlays [1,3,7,8,9,10]. Owing to its exceptional mechanical strength, wear resistance, and biocompatibility, as well as its favorable esthetics, zirconia has become a material of choice for both anterior and posterior restorations [7,8].

Advancements in digital manufacturing technologies, particularly Computer-Aided Design/Computer-Aided Manufacturing (CAD/CAM), have greatly enhanced the efficiency and precision of processing titanium alloys and zirconia in dental laboratories for dental applications [11,12,13]. These technologies encompass both subtractive manufacturing (e.g., milling) and additive manufacturing (e.g., 3D printing), each with distinct advantages and limitations in prosthodontic workflows [11,12,14]. By enabling the fabrication of highly accurate and precise restorations, digital methods minimize manual labor, streamline clinical and laboratory procedures, and reduce overall treatment costs compared to conventional techniques [11,15].

In subtractive manufacturing, titanium and zirconia are processed from prefabricated blocks or discs, where precision milling tools remove excess material to achieve the desired design specifications [11,12]. Zirconia, particularly yttria-stabilized tetragonal zirconia polycrystal (Y-TZP), is widely favored for this method due to its efficient processing speed and cost-effectiveness and the broad availability of compatible materials and dry/wet milling systems [1,3,11,13,16,17]. However, its disadvantages include significant material waste and restricted capability in producing complex geometries [12]. Titanium, including commercially pure titanium (CP Ti) and titanium alloy (Ti-6Al-4V), can also be processed via subtractive methods for various dental applications. However, its high hardness presents certain challenges, such as tool wear and the need for continuous cooling to manage heat generation during milling [1,3,11].

Additive manufacturing (3D printing) is an emerging and promising technique capable of fabricating titanium and zirconia components by building structures layer by layer using various 3D printing technologies [11,14,15]. The additive manufacturing of zirconia has also attracted considerable interest in dental research because of its promising potential for fabricating complex ceramic restorations [11,12]. In 3D printing, zirconia is typically prepared as a ceramic slurry consisting of zirconia powder mixed with a photopolymer resin or binder, which is optimized for appropriate viscosity and flow characteristics. The printing stage is commonly performed using stereolithography (SLA) or digital light processing (DLP) techniques [10,12]. In these methods, the slurry is cured layer by layer using light to form a green (pre-sintered) part [10,12,18]. After printing, the part is dried and then subjected to post-processing through sintering at high temperatures to achieve the necessary strength and durability for clinical dental applications. A significant benefit of 3D printing of zirconia is its material efficiency, as only a small amount of ceramic suspension is required to create the intended object, which helps reduce material waste [11]. Research has demonstrated the efficacy of 3D printing of zirconia for dental restorations [18]. For instance, Lithoz (Vienna, Austria) employs a proprietary light-curing manufacturing (LCM) process specifically tailored for dental-grade zirconia and has successfully fabricated crowns for lower molars using this technology [19]. However, the use of 3D-printed zirconia for producing dental prostheses has not yet become commercially widespread, due to challenges related to the quality and consistency of the final products.

Additive manufacturing of titanium alloys has been successfully implemented using Powder Bed Fusion (PBF) methods such as Laser Sintering (LS), Direct Metal Laser Sintering (DMLS), Selective Laser Melting (SLM), and Selective Electron Beam Melting (SEBM) [4,11,12,14,15,18,20,21]. This technique involves the layer-by-layer fusion of metallic powders using a focused laser beam, guided by Computer-Aided Design (CAD) data. The processing of titanium in this method is highly sensitive to various parameters, including laser type, power settings, spot size, scan speed, layer thickness, build orientation, and powder characteristics such as particle size, shape, and chemical composition [4,11,14]. Recent studies have optimized these parameters, enabling the production of near-fully dense titanium components with improved mechanical properties and structural integrity [14,22].

The combination of titanium-based alloys and zirconia-based ceramics has been increasingly applied in dental prostheses. In such dental restorations, titanium is used for the implant root, abutment, or framework for fixed prostheses, whereas zirconia is used for copings or restorative materials such as crowns and bridges [2,3,6,10,17]. Implant crowns often feature a titanium base combined with a zirconia crown, which is typically screw-retained [17]. In multi-unit prostheses, a two-piece implant crown design is frequently adopted for ease of handling, with components bonded using resin cement [2,17]. Additionally, hybrid abutments comprising titanium substructures and zirconia superstructures are increasingly used and are often bonded using dental cement [3,5,17]. However, susceptibility to catastrophic mechanical failures at the interface between titanium and zirconia-based materials remains a significant challenge [2,3,5]. Although several studies have proposed protocols to enhance the retention strength between these materials, to the best of our knowledge, no prior research has specifically examined the use of different digital fabrication methods for both titanium and zirconia components.

The literature on the adhesion properties of 3D-printed zirconia has shown bonding strength comparable to that of zirconia manufactured via CAD/CAM methods [3,23]. Additionally, one study found that 3D-printed zirconia exhibited superior bond strength compared to milled alternatives [24]. However, the bond strength between titanium and zirconia, fabricated using additive and subtractive techniques, remains insufficiently understood, raising concerns about the long-term durability and clinical performance of titanium–zirconia-based dental prostheses [3]. The objective of this study was to evaluate the bonding strength and durability of titanium alloys fabricated through different digital methods when bonded to zirconia-based materials produced using various digital techniques. The null hypothesis proposes that 3D-printed titanium and zirconia samples will exhibit higher bond strength than their milled counterparts.

## 2. Materials and Methods

### 2.1. Sample Preparation

**Figure 1:**
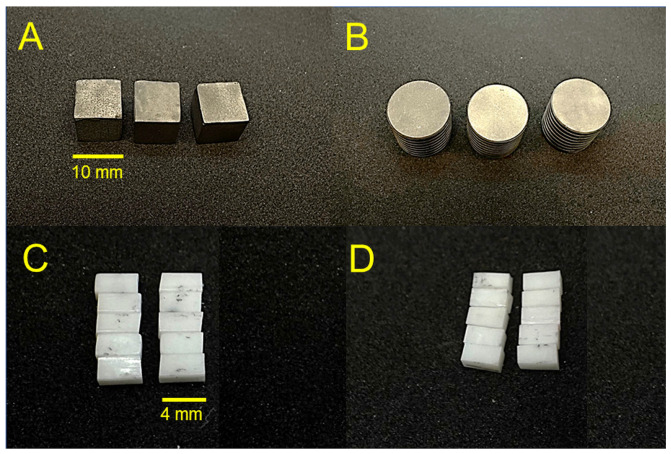
Photographic images of (**A**) 3D-printed titanium specimens (Ti-3D), (**B**) milled titanium specimens (Ti-ML), (**C**) 3D-printed zirconia specimens (Zr-3D), and (**D**) milled zirconia specimens (Zr-ML).

Four groups of digital dental materials were fabricated (Ti-ML, Ti-3D, Zr-ML, and Zr-3D), with 10 specimens from each Ti group and 30 specimens from each Zr group, for a total of 80 specimens (Figure 1). First, two groups of titanium alloys were prepared using two different digital manufacturing methods: CAD/CAM milling method, Ti-ML (N = 10), and CAD/CAM 3D printing method, Ti-3D (N = 10). The specimens for the Ti-ML group were prefabricated titanium discs with dimensions of 20 mm and 50 mm height (Everest T-Blank, KaVo Dental GmbH, Biberach, Germany). The specimens in the Ti-3D group were 3D-printed in a cube shape with dimensions of 10 mm. The specimens in this group were printed using an SLM machine (AM250, Renishaw, UK) with a build envelope volume of 250 mm × 250 mm × 300 mm. The machine was equipped with a 200 W laser with a wavelength of 1070 nm. The process parameters included a layer thickness of 70 µm, laser power of 90 W, point distance of 70 µm, exposure time of 70 µs, and hatching distance of 100 µm. The titanium powder used was Ti6Al4V ELI, with a particle size ranging from 20.6 to 48.6 µm, gas-atomized and spherical [14,21]. The chemical composition of the powder was as follows: Al 6.5%, V 3.9%, Fe 0.2%, O 0.11%, C 0.01%, N 0.03%, H < 0.01%, and Ti balance.

Two groups of zirconia ceramics (N = 60) were prepared using two different digital manufacturing methods: CAD/CAM milling method, Zr-ML (N = 30), and CAD/CAM 3D printing method, Zr-3D (N = 30). For the milled zirconia, a conventional low-translucency material (3 mol% Y-SZ) was used (Ceramill Zi, Amann Girrbach, Koblach, Austria). The specimens were milled by using CAD/CAM technology (Ceramill Motion 2, Amann Girrbach, Koblach, Austria). Specimens were then sintered according to the manufacturer’s recommendations, achieving final dimensions of 4 × 2 × 2 mm (±0.2 mm). The specimens for the Zr-3D group were 3D-printed zirconia (LithaCon 3Y 210, Lithoz, Vienna, Austria) using the DLP/LCM system (Cerafab 7500 Lithoz, Vienna, Austria). After 3D printing, samples were cleaned using Lithasol30 solution and air pressure. Debinding and sintering were performed in a high-temperature furnace (LHTCT 0816; Nabertherm, Germany) following manufacturer-recommended parameters: 25 μm layer height, 110 mJ/cm^2^ DLP energy, and 96.6 mW/cm^2^ light intensity. The samples were manually cleaned, dried at 45 °C, and, unlike other materials, did not undergo UV curing, as per the manufacturer’s instructions. The sintering process for LithaCon 3Y 210 samples involved heating to a maximum temperature of 1600 °C and maintaining this temperature for 2 h. After sintering, the specimens were slowly cooled to room temperature to maintain their structural integrity.

### 2.2. Sample Treatments

All metallic specimens from the Ti-ML and Ti-3D groups underwent surface treatment via sandblasting (airborne-particle abrasion) using a standardized machine (GOBI-2, Wassermann Dental-Maschinen GmbH, Hamburg, Germany). The treatment employed aluminum oxide (Al_2_O_3_) particles with a particle size of 50 µm, at a blasting angle of 45°, for a duration of 15 s, and from a distance of 25 mm. The zirconia specimens from the Zr-ML and Zr-3D groups were left untreated in their as-machined or as-printed state, without sandblasting or further surface modifications.

### 2.3. Density Assessment

The apparent density of ten titanium and zirconia specimens from each group was determined using a gravimetric method adapted from ASTM B212 [25]. Each specimen’s mass (M) was measured with an analytical balance (±0.0001 g accuracy), while its volume (V) was calculated based on its dimensions. The apparent density was then calculated using the formula

Density = Mass/Volume(1)

### 2.4. Microhardness Assessment

A Vickers microhardness indenter (Nova 130; Innovatest Europe BV, Maastricht, The Netherlands) with an indentation force of 500 g and a dwell time of 10 s was employed to measure the microhardness of the polished titanium and zirconia from each group (N = 3). Five indentations at different spots were measured for each specimen, and the average microhardness values (HV) were calculated from the 15 readings for each group.

### 2.5. Surface Roughness Assessment

The surface roughness of both titanium and zirconia specimens was assessed using a non-contact optical profilometer (Contour GT, Bruker, Billerica, MA, USA). The titanium surfaces were sandblasted, while the zirconia surfaces were evaluated in their as-machined or as-printed state, without sandblasting or additional surface modifications. Five distinct measurements for each specimen (N = 3) were taken at different locations with a length of 90 µm, a threshold of 4%, a speed setting of ×2, and a measurement mode of VSL setting. The average surface roughness (Sa) values, expressed in micrometers (µm), were calculated from 15 individual assessments.

### 2.6. Bond Strength Assessment

Two groups of zirconia specimens fabricated using subtractive and additive techniques (Zr-ML and Zr-3D) were bonded to two groups of titanium specimens also fabricated using subtractive and additive techniques (Ti-ML and Ti-3D) using dental cement. This resulted in a total of 48 test samples from four combination groups: (1) Ti-ML/Zr-ML, (2) Ti-ML/Zr-3D, (3) Ti-3D/Zr-ML, and (4) Ti-3D/Zr-3D (N = 12 per group). The titanium specimens, fabricated as cubes or discs with multiple bonding surfaces, allowed several zirconia samples to be bonded to each titanium specimen, one per surface. Before bonding, the specimens were cleaned in an ultrasonic bath and air-dried. A 2 mL layer of self-adhesive resin cement (Totalcem, Itena, Paris, France) was applied to the substrate surfaces, followed by alignment of the specimens with an approximate 1 mm gap between them. Finally, the samples were light-cured (Hilux 250; First Medica, Greensboro, NC, USA) for 60 s, followed by an additional setting period of 5 min.

To assess the aging process of bond strength, half of the specimens (N = 6 per group) from each combination group underwent thermocycling. The specimens were thermocycled using a Huber 1100 unit (SD Mechatronik GmbH, Feldkirchen-Westerham, Germany) through alternating 15 s immersions in water baths set at 5 °C and 55 °C, with a 15 s pause between transitions. The thermocycling process, conducted for 3000 cycles, simulated approximately 2.5 years of oral use [26].

**Figure 2:**
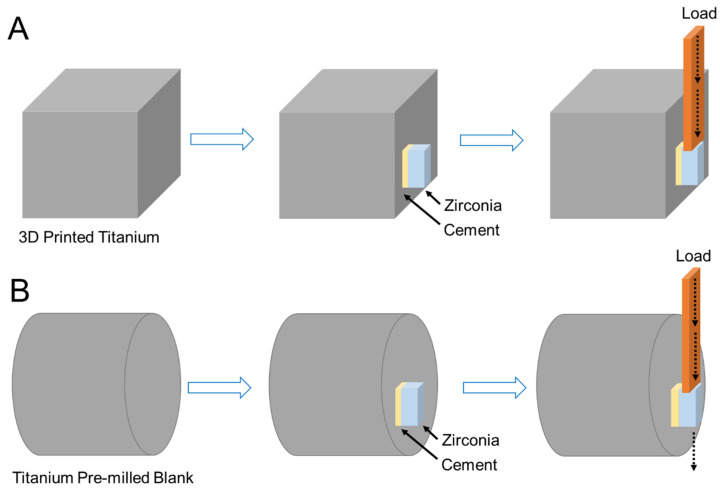
Schematic illustration of the mechanism of the shear bond strength (SBS) test for 3D-printed (**A**) and pre-milled blank (**B**) titanium specimens.

The shear bond strength (SBS) of the samples pre- and post-thermocycling was assessed using a universal testing machine (Instron Corp., MA, USA) at a constant speed of 1.0 mm/min. Force was applied to the zirconia surface until the materials detached from the titanium alloys (Figure 2). The maximum force (Fmax) was measured and used for statistical analysis, with the shear bond strength (SBS) determined using the corresponding formula:

SBS = Fmax/B(2)

where Fmax represents the load at failure in Newtons (N), and B is the bonded surface area (mm^2^).

### 2.7. Failure Mode Assessment

The debonded specimen surfaces from the SBS test were inspected using a stereomicroscope (Nikon SM2-10, Tokyo, Japan) at 15× magnification. The debonded surfaces were categorized into 3 failure patterns: “cohesive failure”, which occurs within the applied selected material; “adhesive failure”, which appears at the interface between the applied selected material and the titanium or zirconia surfaces; and “mixed failure”, indicating a combination of both “adhesive” and “cohesive” failure modes.

### 2.8. Scanning Electron Microscopy

The surfaces of the titanium and zirconia specimens from each group, in their initial state prior to bonding, were examined using scanning electron microscopy (SEM; JEOL, JSM 5900LV, Tokyo, Japan). One specimen from each group was analyzed at an acceleration voltage of 20 kV and a magnification of 100×.

### 2.9. Statistical Analysis

The sample size for the study was determined using G*Power software (v.3.01; Kiel, Germany), according to a pilot study with 80% power, an anticipated effect size of 0.52, and an alpha level of 0.05. Statistical differences between the study groups were analyzed using one-way analysis of variance (ANOVA) and Tukey’s HSD tests for density and shear bond strength (SBS). OriginPro (v.10.2; Origin Lab, Northampton, MA, USA) was used for statistical analysis, with a significance level of α = 0.05.

## 3. Results

The measured physical properties of the titanium and zirconia groups prior to bonding (Table 1) revealed statistically significant differences in the density, microhardness, and surface roughness (*p* < 0.05). The density values showed that the zirconia groups (Zr-ML: 5.81 ± 0.17 g/cm^3^ and Zr-3D: 5.84 ± 0.11 g/cm^3^) had significantly higher densities compared to the titanium groups, with Ti-ML at 4.40 ± 0.10 g/cm^3^ and Ti-3D at 4.08 ± 0.28 g/cm^3^. Among the Ti groups, Ti-ML exhibited a significantly higher density than Ti-3D. However, no significant difference was found in the density between the zirconia groups (Zr-ML and Zr-3D).

The 3D-printed zirconia (1694.2 ± 42.6 HV) showed higher microhardness than the milled zirconia (1238.7 ± 31.6 HV), and the 3D-printed titanium (369.1 ± 5.6 HV) was harder than the milled titanium (219.6 ± 1.4 HV). Overall, both zirconia groups exhibited greater microhardness than all titanium groups, reflecting zirconia’s inherently harder nature.

**Figure 3:**
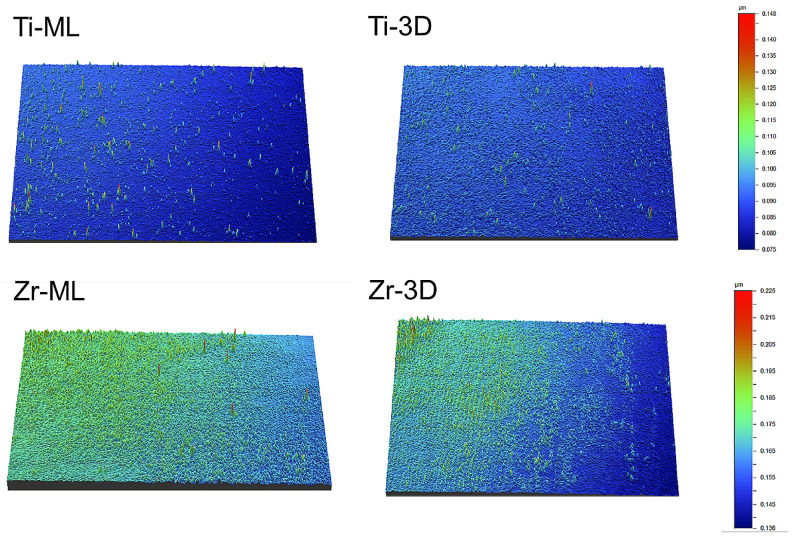
Three-dimensional surface roughness images of the groups, with color variations representing surface depth differences.

Regarding Sa (µm), Zr-ML had the highest value (0.170 ± 0.017 µm), followed by Zr-3D (0.159 ± 0.002 µm), Ti-ML (0.137 ± 0.011 µm), and Ti-3D (0.101 ± 0.001 µm), with each group showing statistically different values (Table 1). Figure 3 shows the 3D surface roughness images of the groups, with color variations indicating differences in surface depth.

The SBS values and failure modes for the titanium/zirconia groups before and after thermocycling are listed in Table 2. Before thermocycling, Ti-ML/Zr-ML showed the highest mean SBS (8.5 ± 2.4 MPa), followed by Ti-ML/Zr-3D (6.6 ± 2.0 MPa) and Ti-3D/Zr-3D (6.8 ± 1.7 MPa), showing significantly higher values than Ti-3D/Zr-ML (5.3 ± 1.9 MPa). After thermocycling, SBS decreased in all groups, with significant reductions in Ti-ML/Zr-3D (4.4 ± 1.2 MPa, *p* = 0.005) and Ti-3D/Zr-ML (2.1 ± 1.2 MPa, *p* < 0.001). Ti-3D/Zr-ML showed the lowest post-thermocycling SBS, while Ti-ML/Zr-ML maintained a higher strength (7.5 ± 1.7 MPa), with no significant reduction compared to its pre-thermocycling value (*p* = 0.501).

**Figure 4:**
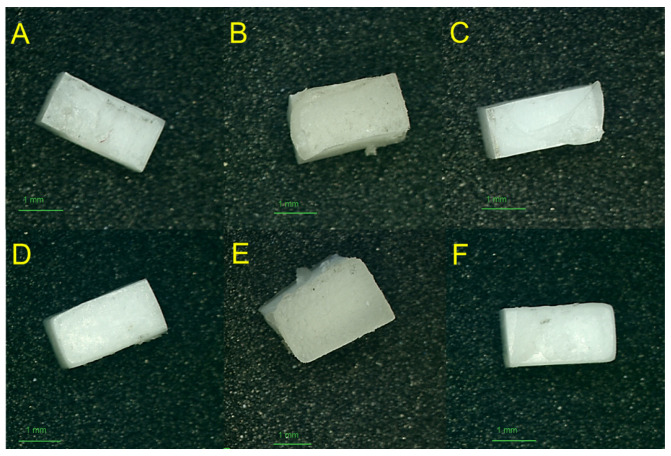
Stereomicroscopic images of zirconia surfaces after debonding from titanium–zirconia test samples, illustrating various failure modes observed across groups: (**A**) adhesive failure, (**B**) cohesive failure, and (**C**) mixed failure in Zr-ML group; (**D**) adhesive failure, (**E**) cohesive failure, and (**F**) mixed failure in Zr-3D group.

Failure mode analysis showed a shift toward cohesive and mixed failures in Ti-ML/Zr-ML after thermocycling, indicating stronger interfacial bonding. The Ti-3D/Zr-ML and Ti-ML/Zr-3D groups showed increased adhesive failure on the zirconia side (AD2), suggesting reduced bond durability after thermal aging (Table 2). Figure 4 presents stereomicroscopic images of the zirconia surfaces after debonding, highlighting the different failure modes and the quality of adhesion observed among the groups.

**Figure 5:**
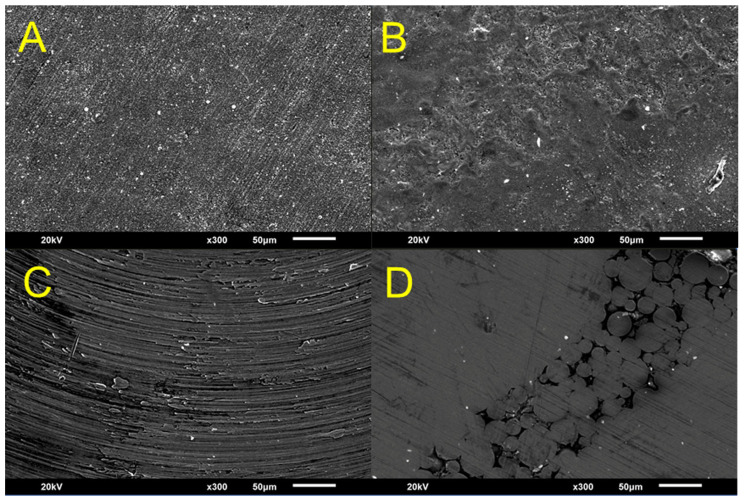
SEM images of zirconia and titanium surfaces prior to bonding, showing distinct surface microstructures among the groups: (**A**) Zr-ML, (**B**) Zr-3D, (**C**) Ti-ML, and (**D**) Ti-3D.

Figure 5 presents scanning electron microscopy (SEM) images of the titanium and zirconia surfaces before bonding. The images show differences in the surface microstructures. Milled titanium (Ti-ML) and zirconia (Zr-ML) surfaces exhibit uniform textures with machining patterns, whereas 3D-printed titanium (Ti-3D) and zirconia (Zr-3D) display irregular topographies with layered patterns.

## 4. Discussion

This study aims to evaluate the bond strength and durability of titanium alloys and zirconia materials fabricated using milling and 3D printing technologies. The null hypothesis proposed that 3D-printed titanium and zirconia samples would exhibit higher bond strength than their milled counterparts. However, the results showed that the combination of milled titanium and milled zirconia exhibited a significantly higher bond strength than the other 3D-printed groups. Therefore, the null hypothesis is rejected.

Despite advancements in digital fabrication, the bond strength between digitally produced titanium and zirconia remains insufficiently explored, raising concerns about the long-term durability and clinical reliability of such material combinations [8,16]. To the best of our knowledge, this study represents the first investigation into the bond strength between titanium and zirconia, where both materials were fabricated via milling and 3D printing technologies.

The capacity to establish sufficient bonding between titanium and zirconia fabricated through subtractive and additive techniques shows potential for dental applications, including implant-supported prostheses, full-arch restorations on metal frameworks, and maxillofacial reconstructions [2,5,6,27]. These bonding techniques are pertinent to medical applications such as orthopedic prostheses and industrial components [1]. This study provides insights into the durability of the titanium–zirconia interface, enhancing the understanding of the clinical feasibility and long-term performance of digital prosthetic components [8,16].

In this study, the physical and mechanical properties of all titanium and zirconia groups were assessed prior to bonding (Table 1), including density, microhardness, and surface roughness, to better understand the factors that may influence bonding performance [5,9]. These parameters were selected based on their known influence on the material performance and interfacial bonding behavior [5]. A higher density typically reflects reduced porosity, which minimizes internal defects and enhances the potential for a uniform stress distribution [20]. Similarly, increased microhardness indicates greater resistance to localized plastic deformation, which may contribute to an improved load-bearing capacity at the bonded interface [28]. Surface roughness is a critical factor for micromechanical retention, as increased surface irregularities can enhance the mechanical interlocking between titanium and zirconia substrates [2,9,13]. The combined effect of these physical properties is essential for optimizing the adhesive interface, ultimately affecting the bond strength and long-term durability under functional loading conditions [9,13]. The mechanical SBS test was selected because of the dominance of shear forces during chewing [3,5,7].

This study demonstrates that the manufacturing methods, whether milling or 3D printing, significantly affect the density of titanium specimens, which might influence their bonding performance [28]. Milled titanium exhibits a higher density, whereas 3D-printed titanium shows a lower density. Notably, research has successfully manipulated the density of 3D-printed titanium by adjusting printing parameters such as layer thickness, build orientation, and laser settings [22]. In contrast, the zirconia samples in this study demonstrated no significant density differences between the two methods, indicating that both milling and 3D printing produce zirconia with comparable bulk properties.

This study demonstrated that both the material type and manufacturing method significantly influenced microhardness, with 3D-printed materials consistently exhibiting higher hardness than their milled counterparts. This finding contrasts with those of previous studies that reported higher hardness in milled objects than in 3D-printed objects [29,30]. The observed enhancement in hardness may be attributed to the improved microstructural homogeneity and fewer internal flaws associated with additive manufacturing [4]. Regardless of the fabrication method, the zirconia groups exhibited significantly higher hardness than the titanium groups, reflecting the inherently harder and more brittle nature of zirconia [13]. These findings highlight the potential of 3D printing for greater design flexibility and enhanced mechanical performance of dental prosthetic materials.

The increased surface roughness of the milled groups was likely due to the milling process, where mechanical cutting tools create micro-irregular textures [23]. In contrast, the Zr-3D group, produced via digital light processing (DLP), exhibited smoother surfaces, as DLP is known for its precision in additive manufacturing [11]. Additionally, the lower surface roughness observed in the 3D-printed groups may be attributed to their microstructural homogeneity and the intrinsic properties of the materials used, resulting in a finer surface finish and reduced topographical irregularities [31].

The SBS results for the Ti-ML/Zr-ML group exhibited the highest bond strength before and after thermocycling, indicating strong and durable adhesion compared to other studies without primers or adhesive agents [2,3,13,27]. In contrast, the Ti-3D/Zr-ML group showed the lowest SBS values, with a significant decrease after thermocycling (*p* < 0.001), likely due to the smoother surface of 3D-printed titanium, which may limit mechanical interlocking [2,17]. This suggests that 3D-printed titanium may be less favorable without optimized surface treatments such as increasing the duration or pressure of airborne-particle abrasion [5,8,17]. Notably, bond strength remained stable in the Ti-ML/Zr-ML and Ti-3D/Zr-3D groups, possibly due to the use of similar fabrication methods, resulting in more compatible and homogenous bonding interfaces. In addition, failure mode analysis supports these observations that the Ti-ML/Zr-ML group showed a shift toward cohesive failures after thermocycling, reflecting stronger interfacial bonding [32]. Conversely, increased adhesive failures in the Ti-3D/Zr-ML and Ti-ML/Zr-3D groups correlate with their reduced bond strength.

The observed adhesion values of 5–8 MPa, achieved without surface treatments, are relatively low but align with those reported in the literature for untreated conditions [33]. Using standard clinical protocols, such as MDP-containing primers or air abrasion, bond strength can be significantly improved to clinically acceptable levels of 12 Mpa [33,34]. This study has some limitations that should be considered. The scope was restricted by factors such as sample size, type of cement, material selection, and milling and 3D printing technology used, which may limit the generalizability of the findings. Furthermore, airborne-particle abrasion was applied to titanium, whereas zirconia remained untreated, which may have influenced bonding outcomes. Additionally, this study focused solely on shear bond strength without assessing other mechanical properties. Future research should explore diverse surface treatments, printing parameters, aging simulations, and clinical trials to validate long-term clinical performance.

## 5. Conclusions

Considering the limitations of this in vitro study, it can be concluded that both the material type and digital fabrication method (milling or 3D printing) significantly influence the physical properties and bonding performance of titanium and zirconia. Milled specimens exhibited higher density in titanium but not in zirconia and demonstrated lower microhardness along with greater surface roughness than their additively manufactured counterparts. Notably, combinations in which both titanium and zirconia were fabricated using the same method demonstrated greater bond durability after thermocycling, likely owing to the improved surface compatibility and homogeneity of the fabrication.

## Figures and Tables

**Table 1:** Density, Vickers microhardness (HV), and surface roughness values (mean ± SD) of the titanium and zirconia groups prior to bonding.

	Group	Density (g/cm^3^)	Microhardness (HV)	Surface Roughness (Sa, µm)
1	Ti-ML	4.40 ± 0.10 ^a^	219.6 ± 1.4 ^a^	0.137 ± 0.011 ^a^
2	Ti-3D	4.08 ± 0.28 ^b^	369.1 ± 5.6 ^b^	0.101 ± 0.001 ^b^
3	Zr-ML	5.81 ± 0.17 ^c^	1238.7 ± 31.6 ^c^	0.170 ± 0.017 ^c^
4	Zr-3D	5.84 ± 0.11 ^c^	1694.2 ± 42.6 ^d^	0.159 ± 0.002 ^d^

Different lowercase letters indicate statistically significant differences between the groups (*p* < 0.05).

**Table 2:** Shear bond strength (SBS) values (mean ± SD) and associated failure modes for titanium–zirconia groups before (Pre) and after (Post) thermocycling.

#	Group	Shear Bond Strength (SBS)			Failure Mode (AD1/AD2/CO/MI)	
		Pre	Post	*p*-Value	Pre	Post
1	Ti-ML/Zr-ML	8.5 ± 2.4 ^a^	7.5 ± 1.7 ^a^	0.501	5/3/2/2	2/1/8/1
2	Ti-ML/Zr-3D	6.6 ± 2.0 ^a^	4.4 ± 1.2 ^b,^ *	0.005	4/2/4/2	1/2/5/4
3	Ti-3D/Zr-ML	5.3 ± 1.9 ^b^	2.1 ± 1.2 ^c,^ *	<0.001	3/2/5/2	2/2/4/4
4	Ti-3D/Zr-3D	6.8 ± 1.7 ^a^	4.6 ± 0.5 ^bd^	0.089	2/3/5/2	2/2/6/2

Different lowercase letters indicate significant differences between groups (*p* < 0.05); * indicates significant differences between pre- and post-thermocycling within the same group (*p* < 0.05). AD1: adhesive failure on the titanium side; AD2: adhesive failure on the zirconia side; CO: cohesive failure; MI: mixed failure mode.

## Data Availability

The original contributions presented in the study are included in the article, further inquiries can be directed to the corresponding author.

## References

[1] Hanawa T. (2020). Zirconia versus titanium in dentistry: A review. Dent. Mater. J..

[2] Nakamura K., Kawaguchi T., Ikeda H., Karntiang P., Kakura K., Taniguchi Y., Toyoda K., Shimizu H., Kido H. (2022). Bond durability and surface states of titanium, Ti-6Al-4V alloy, and zirconia for implant materials. J. Prosthodont. Res..

[3] Serichetaphongse P., Chitsutheesiri S., Chengprapakorn W. (2022). Comparison of the shear bond strength of composite resins with zirconia and titanium using different resin cements. J. Prosthodont. Res..

[4] Wang J., Liu Y., Rabadia C.D., Liang S.-X., Sercombe T.B., Zhang L.-C. (2021). Microstructural homogeneity and mechanical behavior of a selective laser melted Ti-35Nb alloy produced from an elemental powder mixture. J. Mater. Sci. Technol..

[5] Çakır G., Abbasgholızadeh Z.Ş., Aslan Y.U. (2023). In vitro investigation of shear bond strength of titanium alloy bonded to monolithic zirconia prepared via different surface roughening methods using different cements. Clin. Exp. Health Sci..

[6] Curiel-Aguilera F.P., Griffiths G.R., Rossmann J.A., Gonzalez J.A. (2023). Titanium versus zirconia complete arch implant-supported fixed prostheses: A comparison of plaque accumulation. J. Prosthet. Dent..

[7] Zaher A.M., Hochstedler J., Rueggeberg F.A., Kee E.L. (2017). Shear bond strength of zirconia-based ceramics veneered with 2 different techniques. J. Prosthet. Dent..

[8] Shen D., Wang H., Shi Y., Su Z., Hannig M., Fu B. (2023). The effect of surface treatments on zirconia bond strength and durability. J. Funct. Biomater..

[9] Abram A., Staver L., Rojko F., Štukelj R., Klačić T., Kovačević D., Zore A., Bohinc K. (2025). Adhesion of Streptococcus mutans on highly translucent zirconia: Influence of surface properties and polyelectrolyte multilayer coatings. J. Prosthet. Dent..

[10] Rodríguez-Lozano F.J., López-García S., Sánchez-Bautista S., Pérez-López J., Raigrodski A.J., Revilla-León M. (2023). Effect of milled and lithography-based additively manufactured zirconia (3Y-TZP) on the biological properties of human osteoblasts. J. Prosthet. Dent..

[11] Alageel O. (2022). Three-dimensional printing technologies for dental prosthesis: A review. Rapid Prototyp. J..

[12] Wang Y., Zhou Y., Zhu H., Jiang J., He F. (2025). Accuracy, fit, and marginal quality of advanced additively manufactured and milled zirconia 3-unit fixed dental prostheses. J. Prosthet. Dent..

[13] Hansson M., Ågren M. (2024). Shear bond strength of adhesive cement to zirconia: Effect of added proportion of yttria for stabilization. J. Prosthet. Dent..

[14] Alageel O., Alfrisany N., Aldosari A., Qashish S., Alsarani M.M., AlFaify A.Y. (2024). Impact of density variations and growth direction in 3D-printed titanium alloys on surface topography and bonding performance with dental resins. Crystals.

[15] Alageel O., Abdallah M.N., Alsheghri A., Song J., Caron E., Tamimi F. (2018). Removable partial denture alloys processed by laser-sintering technique. J. Biomed. Mater. Res. B Appl. Biomater..

[16] Sokolowski G., Szczesio-Wlodarczyk A., Szynkowska-Jóźwik M.I., Stopa W., Sokolowski J., Kopacz K., Bociong K. (2023). The shear bond strength of resin-based luting cement to zirconia ceramics after different surface treatments. Materials.

[17] Bergamo E.T., Zahoui A., Ikejiri L.L.A., Marun M., da Silva K.P., Coelho P.G., Soares S., Bonfante E.A. (2021). Retention of zirconia crowns to Ti-base abutments: Effect of luting protocol, abutment treatment and autoclave sterilization. J. Prosthodont. Res..

[18] Li R., Xu T., Wang Y., Sun Y. (2023). Accuracy of zirconia crowns manufactured by stereolithography with an occlusal full-supporting structure: An in vitro study. J. Prosthet. Dent..

[19] Schweiger J., Bomze D., Schwentenwein M. (2019). 3D printing of zirconia–what is the future?. Curr. Oral Health Rep..

[20] Alageel O., Alhijji S., Alsadon O., Alsarani M., Gomawi A.A., Alhotan A. (2023). Trueness, flexural strength, and surface properties of various three-dimensional (3D) printed interim restorative materials after accelerated aging. Polymers.

[21] Zluhan B., Narasimharaju S.R., Cholkar A., Thomas K., Raghavendra R., Lopes E.S. (2025). Design, defect analysis, compressive strength and surface texture characterization of laser powder bed fusion processed Ti6Al4V lattice structures. J. Mater. Res. Technol..

[22] Alfaify A.Y., Hughes J., Ridgway K. (2018). Critical evaluation of the pulsed selective laser melting process when fabricating Ti64 parts using a range of particle size distributions. Addit. Manuf..

[23] Moon J.M., Jeong C.S., Lee H.J., Bae J.M., Choi E.J., Kim S.T., Park Y.B., Oh S.H.A. (2022). A comparative study of additive and subtractive manufacturing techniques for a zirconia dental product: An analysis of the manufacturing accuracy and the bond strength of porcelain to zirconia. Materials.

[24] Lu Y., Mei Z., Lou Y., Yue L., Chen X., Sun J., Wan Z., Yu H. (2020). Schwickerath adhesion tests of porcelain veneer and stereolithographic additive-manufactured zirconia. Ceram. Int..

[25] (2013). Standard Test Method for Apparent Density of Free-Flowing Metal Powders Using the Hall Flowmeter Funnel.

[26] Wang Y., Huang H., Lin H., Jiang L., Pan Y., Li X., Cheng H. (2020). The influence of recycling on the properties of interface between ceramic and dental alloys. BioMed Res. Int..

[27] Yilmaz B., Gouveia D., Schimmel M., Lu W.-E., Özcan M., Abou-Ayash S. (2024). Effect of adhesive system, resin cement, heat-pressing technique, and thermomechanical aging on the adhesion between titanium base and a high-performance polymer. J. Prosthet. Dent..

[28] Sathiya Narayanan N., Sai Venkat Mohan D., Abhinay J., Dinesh T., Satya Sai Surya Teja V., Praneeth R. (2024). Effects on microhardness, tensile strength, deflection, and drop weight impact resistance with the addition of hybrid filler materials for enhancing GFRP composites. Sci. Rep..

[29] Park Y., Kim J., Kang Y.-J., Shim E.-Y., Kim J.-H. (2024). Comparison of fracture strength of milled and 3D-printed crown materials according to occlusal thickness. Materials.

[30] Cherneva S., Petrunov V., Petkov V., Bogdanov V., Simeonova S. (2023). Structure and mechanical properties of milled and 3D-printed Ti-6Al-4V alloys for subtractive and additive CAD/CAM manufacturing in dentistry. Appl. Sci..

[31] Hashmi A.W., Mali H.S., Meena A. (2023). Improving the surface characteristics of additively manufactured parts: A review. Mater. Today Proc..

[32] Miljkovic M., Dacic S., Mitic A., Petkovic D., Andjelkovic-Apostolovic M. (2022). Shear bond strength and failure modes of composite to dentin under different light-curing conditions. Polym. Polym. Compos..

[33] Salem R.S.T., Ozkurt-Kayahan Z., Kazazoglu E. (2019). In vitro evaluation of shear bond strength of three primer/resin cement systems to monolithic zirconia. Int. J. Prosthodont..

[34] Qeblawi D.M., Campillo-Funollet M., Muñoz C.A. (2015). In vitro shear bond strength of two self-adhesive resin cements to zirconia. J. Prosthet. Dent..

